# Shear hardening in frictionless amorphous solids near the jamming transition

**DOI:** 10.1093/pnasnexus/pgad047

**Published:** 2023-02-10

**Authors:** Deng Pan, Fanlong Meng, Yuliang Jin

**Affiliations:** CAS Key Laboratory of Theoretical Physics, Institute of Theoretical Physics, Chinese Academy of Sciences, Beijing 100190, China; CAS Key Laboratory of Theoretical Physics, Institute of Theoretical Physics, Chinese Academy of Sciences, Beijing 100190, China; School of Physical Sciences, University of Chinese Academy of Sciences, Beijing 100049, China; Wenzhou Institute, University of Chinese Academy of Sciences, Wenzhou, Zhejiang 325000, China; CAS Key Laboratory of Theoretical Physics, Institute of Theoretical Physics, Chinese Academy of Sciences, Beijing 100190, China; School of Physical Sciences, University of Chinese Academy of Sciences, Beijing 100049, China; Wenzhou Institute, University of Chinese Academy of Sciences, Wenzhou, Zhejiang 325000, China

**Keywords:** shear hardening, jamming transition, critical scaling, elasticity theory, amorphous solid

## Abstract

The jamming transition, generally manifested by a rapid increase of rigidity under compression (i.e. compression hardening), is ubiquitous in amorphous materials. Here we study shear hardening in deeply annealed frictionless packings generated by numerical simulations, reporting critical scalings absent in compression hardening. We demonstrate that hardening is a natural consequence of shear-induced memory destruction. Based on an elasticity theory, we reveal two independent microscopic origins of shear hardening: (i) the increase of the interaction bond number and (ii) the emergence of anisotropy and long-range correlations in the orientations of bonds—the latter highlights the essential difference between compression and shear hardening. Through the establishment of physical laws specific to anisotropy, our work completes the criticality and universality of jamming transition, and the elasticity theory of amorphous solids.

**Significance Statement**

Under isotropic compression, a packing of spheres undergoes a jamming transition, at which rigidity emerges. Upon further compression, the rigidity increases due to the growth of inter-particle contact number—this phenomenon is called compression hardening, which can be statistically described by critical scaling laws. Here we investigate the anisotropic counterpart of compression hardening—shear hardening near the jamming transition in frictionless packings. We discover new jamming scaling laws specific to shear deformations from simulation data, and reveal microscopic origins of shear hardening by an elasticity theory. These results will be useful for understanding the mechanisms of related phenomena in granular materials and colloidal suspensions, including the dilatancy effect and shear jamming.

##  

The jamming transition occurs in a wide range of soft materials, ranging from granular matter to colloidal suspensions to glasses. As a non-equilibrium, athermal phase transition, its criticality is specified by a set of scaling laws (1–4). In particular, the scaling relationship (under the harmonic approximation of the inter-particle interaction) *G* ∼ Δ*Z*, between the shear modulus *G* of the jammed phase and the excess coordination number Δ*Z*, has been derived by the mean-field replica glass theory in infinite dimensions (5) and microscopic elasticity theories (6, 7), with a promising agreement to data from isotropic compression simulations (2). Here we demonstrate that this relationship cannot fully account for the shear hardening behavior—the influence of fabric anisotropy (quantified by the macroscopic friction coefficient *μ* = *σ*/*P*, which is the ratio of shear stress *σ* to pressure *P*) is significant. We report numerically a new scaling law unique to shear, *μ* ∼ *σ*^*β*^ with *β* ≈ 0.25, between *μ* and *σ*, and derive theoretically an additional anisotropic contribution to the elasticity, *G*_AI_ ∼ *μ*^2^, verified by our simulation data.

*Shear hardening*, meaning that *G* increases by shear, is closely related to several other interesting phenomena. The vestige of shear hardening in the unjammed phase is the phenomenon of *shear jamming* (the onset of rigidity under a fixed-volume shear deformation) observed in many experiments (8–12) and simulations (13–26). Shear hardening is generally accompanied by increasing pressure under fixed-volume conditions, which implies that under fixed-pressure conditions a *dilatancy effect* would occur (27–29). Recent studies have shown the possibility of complete decoupling between friction and shear jamming/dilatancy (30, 17, 20, 21). In this study, we show that friction is also not essential for shear hardening. Due to the lack of internal friction, the conventional sawtooth model (31) cannot be applied anymore, and frictionless mechanisms are demanded. To this end, here we provide a phenomenological explanation based on the generalized jamming phase diagram (21), and reveal microscopic origins of frictionless shear hardening from the elasticity theory of amorphous solids (32–35, 7, 6). Our simulation and theoretical results demonstrate that fabric anisotropy makes important contributions to shear hardening, which resembles a similar connection between anisotropy and shear jamming (15). Note that the discussed shear hardening occurs in the solid phase (above jamming), which shall not be confused with *shear thickening* that presents in non-Newtonian fluids (below jamming) such as dense suspensions (36).

Shear hardening has been directly observed in previous studies (12, 22, 37, 38). Simulations report a hardening regime on the stress–strain curve, following elastic and softening regimes (37, 22). These previous studies (37, 22) consider mechanically trained packings, which have effectively a moderate degree of annealing. The shear hardening scaling regime in such systems is limited (about one decade) due to the interruption of yielding (note that the yield stress *σ*_Y_ vanishes in the rapid quench limit (39)), making a reliable estimation of the scaling exponents difficult. To overcome this issue, here we simulate ultra-stable packings annealed by an efficient swap algorithm (40–44). The yield stress of our ultra-stable packings is significantly larger than that of mechanically trained packings (38), and consequently the shear hardening scaling regime is extended up to about eight decades. This advantage allows us to accurately determine shear hardening exponents, which turn out to be independent of the degree of annealing.

## Results

### Conditions for shear hardening

**Fig. 1.**
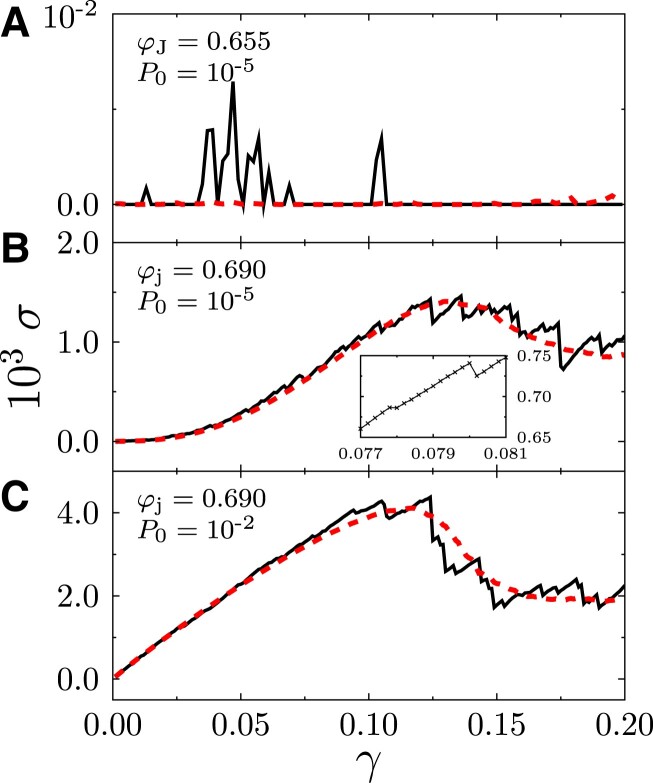
Stress–strain curves. Plotted are stress–strain curves of A) a rapidly quenched system near the jamming transition, B) a deeply annealed system near the jamming transition, and C) a deeply annealed system over-compressed well above the jamming transition. Data are obtained from constant-volume AQS simulations of 3D frictionless soft spheres (SSs). The dashed and solid lines represent averaged and single-realization curves respectively. The inset in panel B) is an enlarged view of the single-realization curve.

As demonstrated by the linear plots in Fig. 1, the stress–strain curves, averaged over samples, typically display three kinds of behavior under constant-volume athermal quasi-static shearing (AQS). (i) Rapidly quenched systems with the minimum jamming density (see Materials and Methods), φ_j_ ≈ φ_J_, behave like *plastic flows* in the zero pressure limit *P*_0_ → 0, where *P*_0_ ≡ *P*(*γ* = 0) is the pressure of unstrained configurations: *σ*(*γ*) ≈ 0 and *G*(*γ*) ≈ 0 (see Fig. 1A). On the stress–strain curve of an individual sample obtained in a single realization of simulation, the coexistence of solid-like (*σ* > 0 and *G* > 0) and liquid-like (*σ* = *G* = 0) states can be identified (39). (ii) Shear hardening, manifested in the increasing function of *G*(*γ*), appears in deeply annealed systems with φ_j_ ≫ φ_J_, at small *P*_0_ (see Fig. 1B). Note that in this case, there is a narrow *shear softening* regime (i.e. *G*(*γ*) is a descending function) before shear hardening, which can only be identified in log scales (see Fig. 3 and Supplementary Material, Fig. S1). (iii) If the systems are over-compressed well above the jamming transition, then shear hardening disappears and only shear softening is observed independent of φ_j_ (see Fig. 1C).

**Fig. 2.**
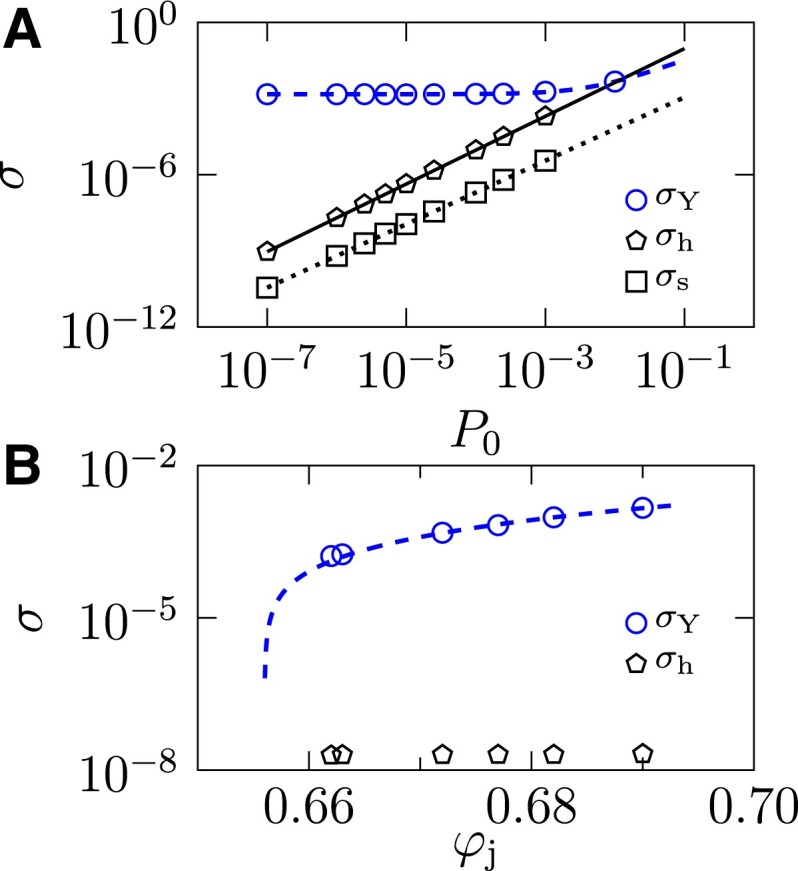
Dependence of several characteristic stresses on *P*_0_ and φ_j_. A) The yielding stress *σ*_Y_, the onset stress *σ*_h_ of shear hardening, and the onset stress *σ*_s_ of shear softening are plotted as functions of the unstrained pressure *P*_0_, with a fixed φ_j_ = 0.69. The dashed line is the fitting curve *σ*_Y_(*P*_0_) = 1.48 × 10^−3^ + 0.317 *P*_0_. The solid and dotted lines are the relations $\sigma_{h}(P_{0})=2.0\times P_{0}^{4/3}$ and $\sigma_{s}(P_{0})=0.02\times P_{0}^{5/4}$ (see Fig. 4). The two lines *σ*_Y_(*P*_0_) and *σ*_h_(*P*_0_) intersect at $P_{0}^{*}=1.13\times 10^{-2}$, above which shear hardening is non-observable. B) The φ_j_-dependence of *σ*_Y_ and *σ*_h_, with a fixed *P*_0_ = 10^−6^. The dashed line is the fitting curve *σ*_Y_(φ_j_) = 0.0175 (φ_j_ − φ_J_) + 0.736 (φ_j_ − φ_J_)^2^, where φ_J_ ≈ 0.655. It is known that *σ*_Y_ vanishes linearly with φ_j_ − φ_J_ in the vicinity of φ_J_ (21); here a quadratic correction is added to account for the deviation from this linear critical scaling in the large φ_j_ regime.

From the above analysis, one finds that shear hardening emerges near the jamming transition (*P*_0_ → 0) in deeply annealed packings (φ_j_ ≫ φ_J_), under constant-volume AQS. These two conditions are demonstrated more quantitatively in Fig. 2. The shear hardening behavior can be only observed in a stress window *σ*_h_ < *σ* < *σ*_Y_, where *σ*_h_ is the onset stress of hardening (see below for how *σ*_h_ is determined). For a fixed φ_j_ with increasing *P*_0_, *σ*_h_ increases faster than *σ*_Y_, as shown in Fig. 2A. Above $P_{0}^{*}$, where $\sigma_{h}(P_{0}^{*})\approx \sigma_{Y}(P_{0}^{*})$, the shear hardening regime disappears. For a fixed *P*_0_, *σ*_h_ is nearly independent of φ_j_ (see Fig. 2B), while *σ*_Y_ decreases with decreasing φ_j_ and vanishes when φ_j_ → φ_J_ (45, 39) (a more detailed study on *σ*_Y_(φ_j_) can be found in Ref. (21)). Thus the scaling regime of shear hardening also vanishes as φ_j_ → φ_J_. The finite-size effects are discussed in Supplementary Material, Sec. 5 (see Fig. S5). In this study, we do not consider other factors, such as finite temperatures or finite shear rates (46).

**Fig. 3.**
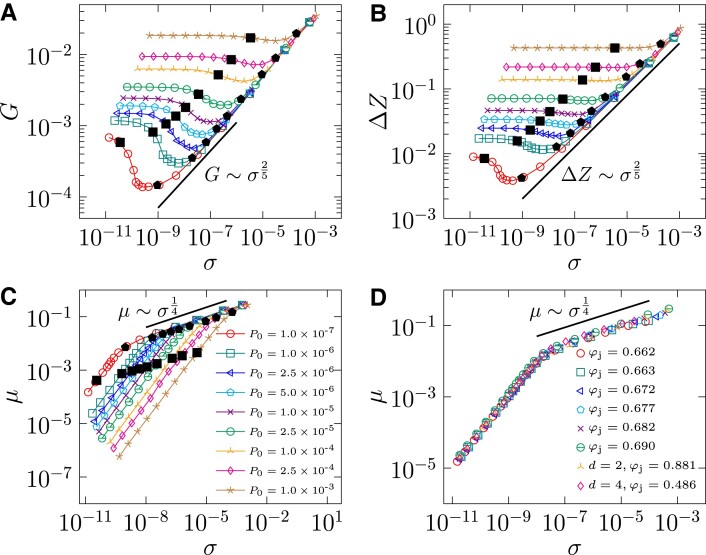
Shear hardening scalings. Simulation results in 3D of A) the shear modulus *G*, B) the excess coordination number Δ*Z* and C) the macroscopic friction coefficient *μ* as functions of stress *σ*, for a few different *P*_0_. The solid lines represent scaling laws in the shear hardening regime, *G* ∼ *σ*^2/5^, Δ*Z* ∼ *σ*^2/5^ and *μ* ∼ *σ*^1/4^. The crossovers at *σ*_s_ and *σ*_h_, which are determined in Fig. 4, are marked by black solid squares and pentagons respectively. D) Universal relationship between *μ* and *σ* in 3D samples prepared by cyclic shear (φ_j_ = 0.662), cyclic compression (φ_j_ = 0.663), and swap thermal annealing (φ_j_ = 0.672, 0.677, 0.682, 0.690), as well as in two and four dimensions (*P*_0_ = 10^−6^ in all cases).

### Critical scaling laws

Below we focus on deeply annealed samples with a large jamming density φ_j_ = 0.69, which are quenched from dense equilibrium liquids at φ_eq_ = 0.643. Based on the log–log plots in Fig. 3, we identify consecutively (before yielding) the elastic, shear softening and shear hardening regimes, separated by two crossover points at *σ*_s_ and *σ*_h_.

**Fig. 4.**
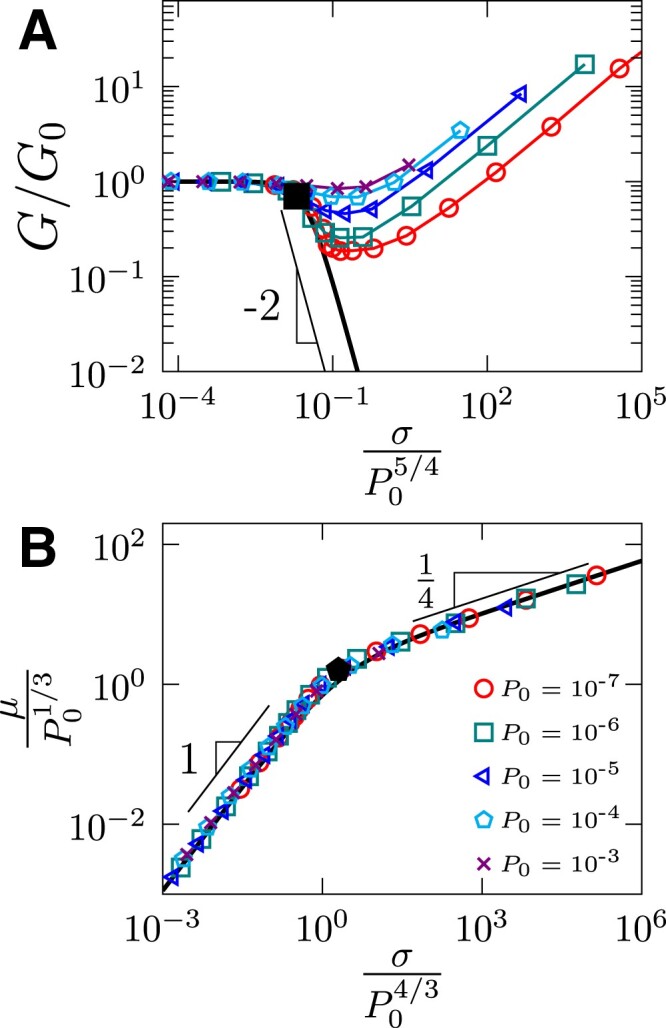
Data collapsing in shear softening and hardening regimes. Rescaled plots of A) shear modulus *G* and B) macroscopic friction *μ* as functions of stress *σ*, where *P*_0_ and *G*_0_ are unstrained pressure and shear modulus. The crossover stresses are numerically defined as $\sigma_{s}/P_{0}^{5/4}=2\times 10^{-2}$ (solid black square) in A) and $\sigma_{h}/P_{0}^{4/3}=2$ (solid black pentagon) in B). The bold lines represent the master curves $y=\frac{1}{1+(32.7x{)}^{2}}$ and $y=\frac{1.13x}{1+(0.52x{)}^{0.75}}$ in A) and B), respectively.

The data of shear modulus in the elastic and shear softening regimes can be described by a scaling function $G(P_{0},\sigma )/G_{0}=G(\sigma /\sigma_{s})$, where *G*_0_ = *G*(*P*_0_, 0) is the unstrained shear modulus, G(x→0)=1, $G(x\to \infty )=x^{-2}$ and $\sigma_{s}∼P_{0}^{5/4}$ (see Fig. 4A). The scaling function G(x) was initially proposed in Ref. (47) to describe both linear and softening behavior in rapidly quenched packings. We find that G(x) agrees well with our data of deeply annealed systems in the linear and softening regimes, but deviate from the data starting from *σ* = *σ*_h_ which is the onset point of shear hardening. The scaling function G(x) allows us to determine numerically the crossover stress $\sigma_{s}=cP_{0}^{5/4}$, where the prefactor *c* is chosen to be 0.02. The *σ*_s_ estimated in this way (squares) reasonably separates linear and softening behavior, as can be seen in Fig. 3A. From G(x), the scaling behavior of linear and softening regimes can be extracted. In the linear regime (*σ* ≪ *σ*_s_ or *x* = *σ*/*σ*_s_ → 0), G(x→0)=1, which means that the shear modulus is simply a constant *G* = *G*_0_. In the softening regime (*σ* ≫ *σ*_s_ or *x* → ∞), *G*(*x* → ∞) = *x*^−2^ which means that *G* ∼ *P*_0_*γ*^−2/3^ (where we have used the well know scaling $G_{0}∼P_{0}^{1/2}$ for un-strained systems (2)). These results are fully consistent with the findings in Ref. (47). Our data show that the presence of a strong shear hardening effect does not change the scalings in linear and softening regimes. It is of interest to provide a theoretical explanation of the softening exponent −2 in *G*(*x* → ∞) = *x*^−2^ in future studies.

The deviation of the simulation data from the master curve G(x) in Fig. 4 is followed by the shear hardening behavior. The scaling of *σ*_h_ is determined based on the rescaled plot of macroscopic friction *μ* in Fig. 4B, with a scaling function $\mu (P_{0},\sigma )/\mu_{h}=U(\sigma /\sigma_{h})$, where U(x→0)∼x, $U(x\to \infty )∼x^{1/4}$, $\sigma_{h}∼P_{0}^{4/3}$ and $\mu_{h}∼P_{0}^{1/3}$. Fig. 4B shows that elastic and softening data follow a universal scaling *μ* ∼ *σ*. Previously, Kawasaki and Miyazaki (22) report two scalings *μ* ∼ *γ* and *μ* ∼ *γ*^1/2^, respectively, in the elastic and softening regimes for a two-dimensional model, which, together with the shear softening scaling *σ* ∼ *γ*^1/2^, give a consistent result *μ* ∼ *σ*.

In the shear hardening regime, *σ*_h_ < *σ* < *σ*_Y_, the system’s behavior is characterized by two scaling laws. Numerical fitting gives *μ* ∼ *σ*^*β*^ with *β* = 0.248 ± 0.006 and Δ*Z* ∼ *σ*^*ν*^ with *ν* = 0.411 ± 0.005 in the shear hardening regime (Fig. 3). The scaling of Δ*Z* is consistent with the ansatz Δ*Z* ∼ *σ*^2/5^ proposed in Ref. (4). Note that this scaling is examined in (4) by looking at the stress variance *σ*^2^ of isotropically compressed configurations with φ_j_ = φ_J_, where the mean stress is zero. Here we provide direct verification of the ansatz, thanks to the emergence of shear hardening in deeply annealed packings (φ_j_ ≫ φ_J_). The scaling *μ* ∼ *σ*^1/4^ (or equivalently *P* ∼ *σ*^3/4^), is absent in the framework of Ref. (4), and to our knowledge, has never been reported previously. Based on the elasticity theory (see below for details), we derive that *G* is a linear combination of *G*_I_ ∼ Δ*Z* and *G*_AI_ ∼ *μ*^2^ , which, together with the above two scalings, gives *G* ∼ *σ*^2/5^ in the jamming limit where *σ* → 0 (see Fig. 3A for a comparison to the numerical data). While it is straightforward to translate these scalings, *G* ∼ Δ*Z* ∼ *σ*^2/5^ and *μ* ∼ *σ*^1/4^, from the stress-dependent forms into strain-dependent forms based on the relation *G* = *dσ*/*dγ*, one should be cautious about the influence of plasticity in the measurement of stress and modulus (47).

It can be shown that the shear hardening scaling exponents are independent of the preparation method (thermal annealing or mechanical training), the degree of annealing represented by φ_j_, and the dimensionality in *d* = 2, 3, 4 dimensions (see Supplementary Material, Figs. S2, S3, and S4). In particular, the data of *μ* versus *σ* collapse remarkably without any shifting (see Fig. 3D).

### Shear hardening as a consequence of memory loss by shear

To understand the origin of shear hardening, it is useful to examine the evolution of state on the generalized jamming phase diagram (21). The key point is that, for a general φ_j_, the isotropic unstrained packings and the asymptotic stationary states at large strains satisfy different sets of equations of states (EOSs); they coincide only when φ_j_ → φ_J_ as in the initial proposal of the phase diagram by Liu and Nagel (45). The pressure, excess coordination number and macroscopic friction of unstrained states are described by the following EOSs, respectively, *P*_0_ ∼ φ − φ_j_, Δ*Z*_0_ ∼ (φ − φ_j_)^1/2^, and *μ*_0_ = 0. The corresponding EOSs of stationary states are *P*_s_ ∼ φ − φ_J_, Δ*Z*_s_ ∼ (φ − φ_J_)^1/2^, and *μ*_c_ − *μ*_s_ ∼ (φ − φ_J_)^1/2^, where *μ*_c_ ≈ 0.1 (21, 48, 49).

**Fig. 5.**
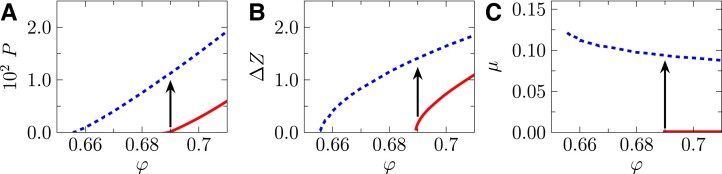
Equations of states. Plotted are equations of isotropically jammed (*γ* = 0, solid lines) and steady states (*γ* = ∞, dashed lines), where the A) pressure *P*, B) excess coordination number Δ*Z*, and C) macroscopic friction coefficient *μ* are plotted as functions of volume fraction φ. The vertical arrows present the constant-volume shear protocol.

Under constant-volume shear, it is easy to see from Fig. 5 that the system has to gain rigidity, because both the pressure and the number of contacts increase. The increase of anisotropy *μ* makes an additional contribution to the shear modulus, as demonstrated below. Shear hardening is thus a natural consequence of the memory erasing process caused by shear. Different unstrained states must evolve to the same stationary state that is independent of the initial condition. Initial states that are dense packings (φ_j_ ≫ φ_J_) created by deep annealing should also be brought back by shear to the generic packings at φ_J_ (corresponding to the rapid quench limit (2))—this process is accompanied by the increase of pressure and shear modulus under the constant-volume condition.

### Microscopic origins of shear hardening

**Fig. 6.**
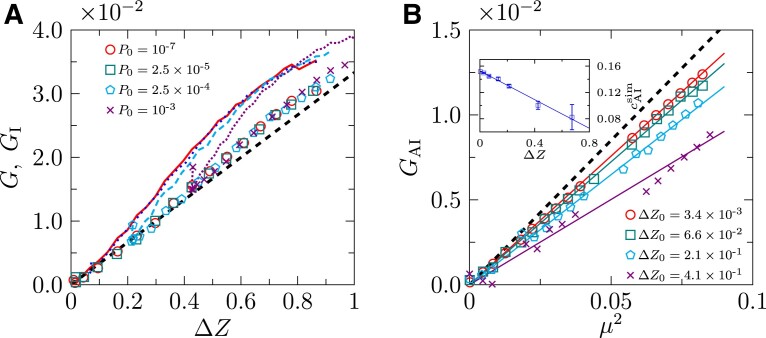
Decomposition of the shear modulus into isotropic and anisotropic parts. A) Total shear modulus *G* (lines) and its isotropic part *G*_I_ (points) as functions of Δ*Z*. The bold dashed line corresponds to the theoretical result *G*_I_ = *c*_I_Δ*Z* with *c*_I_ = 1/30 (7, 35). B) Anisotropic part of the shear modulus *G*_AI_ as a function of *μ*^2^ for a few fixed Δ*Z* = Δ*Z*_0_ (see the legend in (A) for corresponding *P*_0_ values). The bold dashed line indicts our theoretical result *G*_AI_ = *c*_0_*μ*^2^ for Δ*Z* → 0, with estimated *c*_0_ ≈ 0.17. The solid lines represent linear fitting to the data. The slopes are plotted in the inset, which are fitted to a linear function according to Eq. 4, giving $c_{\mathrm{AI}}^{\mathrm{sim}}(\Delta Z)=0.15-0.11\Delta Z$ (line).

Previous studies have developed an elasticity theory for athermal disordered solids near the jamming transition, attributing compression hardening to the increase of interaction bonds via the relation (6, 7, 35),


(1) $$G_{I}=c_{I}\Delta Z,$$

where *c*_I_ = 1/30 (we have set both the bond stiffness *κ* and the unstressed bond length *r*_0_ = *D* to one). While this theoretical result agrees well with the simulation data of sphere packings under isotropic compression (7), it cannot fully account for our shear hardening data (see Fig. 6A).

**Fig. 7.**
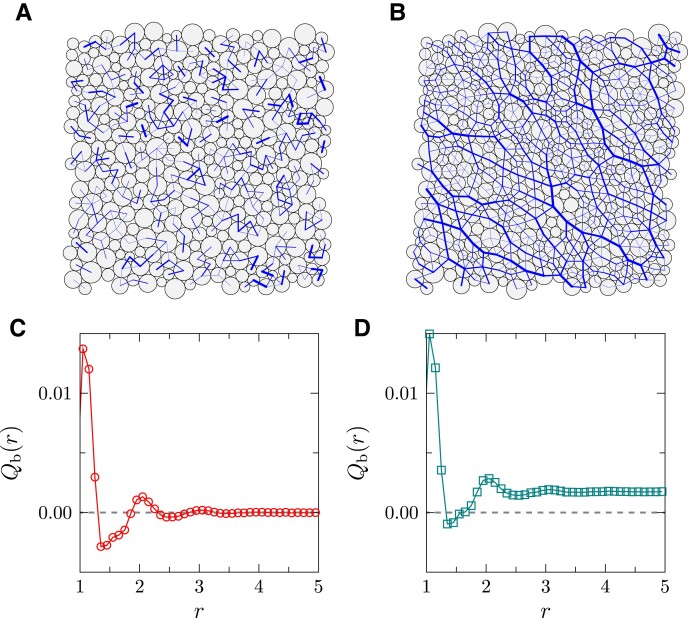
Anisotropy and long-range orientational correlations of contact bonds. A,B) Typical force networks of A) isotropically compressed (*P*_0_ = 10^−6^, *μ* = 0) and B) sheared samples (*P*_0_ = 10^−6^, *μ* = 0.36) in two dimensions, where the width of bond is proportional to the magnitude of contact force. C,D) Bond orientation correlation function *Q*_b_(*r*) of C) isotropically compressed (*P*_0_ = 10^−7^, *μ* = 0) and D) sheared (*P*_0_ = 10^−7^, *μ* = 0.29) 3D packings: *Q*_b_(*r* → ∞) = 0 in C) and *Q*_b_(*r* → ∞) > 0 in D).

To understand the microscopic origin of this deviation, we consider a generalized formula (6) (see Supplementary material for details),


(2) $$G=G_{I}+G_{\mathrm{AI}},$$

for *P* → 0, where $G_{\mathrm{AI}}\approx \frac{1}{N_{\mathrm{NR}}}\sum_{b_{1}\neq b_{2}}^{N_{b}}{\tilde{\;f}}_{b_{1}}{\tilde{\;f}}_{b_{2}}n_{b_{1}}^{x}n_{b_{1}}^{z}n_{b_{2}}^{x}n_{b_{2}}^{z}$ depends on the normalized forces ${\tilde{\;f}}_{b}$ and bond orientations (represented by unit vectors ${\vec{n}}_{b}$), *N*_b_ is the number of contacts and *N*_NR_ is the number of non-rattler particles (rattlers have fewer than *d* + 1 contacts in *d* dimensions). In isotropic packings, the lack of long-range spatial correlations between bond orientations, $\langle n_{b1}^{x}n_{b1}^{z}n_{b2}^{x}n_{b2}^{z}\rangle \approx \langle n_{b1}^{x}n_{b1}^{z}\rangle \langle n_{b2}^{x}n_{b2}^{z}\rangle =0$, suggests that *G*_AI_ ≈ 0 (note that $G_{\mathrm{AI}}\approx \langle {\tilde{\;f}}_{b1}{\tilde{\;f}}_{b2}\rangle \langle n_{b1}^{x}n_{b1}^{z}n_{b2}^{x}n_{b2}^{z}\rangle$ under the effective media approximation (7), and all contact forces ${\tilde{\;f}}_{b}$ are positive). In sheared packings, the bonds tend to align in shear-preferred directions and are orientationally long-range correlated (16): the correlation function, $Q_{b}(r)=\langle n_{0}^{x}n_{0}^{z}n_{b}^{x}n_{b}^{z}\delta (|{\vec{r}}_{0}-{\vec{r}}_{b}|-r)\rangle$, where ${\vec{r}}_{b}$ is the position of bond *b*, decays to a finite value at large *r* (see Fig. 7). Consequently, *G*_AI_ is non-zero in such a case.

Our theoretical analysis predicts a simple relation between *G*_AI_ and the macroscopic friction *μ*,


(3) $$G_{\mathrm{AI}}=c_{\mathrm{AI}}(\Delta Z)\mu^{2},$$

where


(4) $$c_{\mathrm{AI}}(\Delta Z)=c_{0}-\alpha \Delta Z,$$

with two constants *c*_0_ and *α*. In the jamming limit (Δ*Z* ≈ 0), the coefficient *c*_0_ can be evaluated from the formula, $c_{0}=\frac{\langle f\rangle^{2}}{3\langle f^{2}\rangle }$, where 〈*f*〉 and 〈*f*^2^〉 are the first two moments of the force distribution. In our system, we obtain from simulations $\frac{\langle f\rangle^{2}}{\left\langle f^{2}\right\rangle }\approx 0.5$, which gives *c*_0_ ≈ 0.17 (see Supplementary Material, Fig. S6). Above jamming (Δ*Z* > 0), *G*_AI_ is lowered by a higher-order correction term ∼Δ*Zμ*^2^.

To examine Eq. 3, we separate isotropic and anisotropic moduli in our simulation data as follows. Considering that Δ*Z*(*γ*) is increased during shear, at each *γ* we first decompress the configurations keeping *γ* unchanged, until Δ*Z* reaches Δ*Z*_0_ ≡ Δ*Z*(*γ* = 0) that is fixed by the initial condition $\Delta Z_{0}∼P_{0}^{1/2}$. The decompressed configurations at different *γ* thus now have the same Δ*Z* = Δ*Z*_0_, but different *μ* (we denote these configurations as a *constant-*Z* ensemble*). In this way, we remove the contributions from the change of Δ*Z*, left with *G*_AI_ that changes with *μ*. Fig. 6B shows that *G*_AI_ is proportional to *μ*^2^ for any constant Δ*Z*, as predicted by Eq. 3. The behavior of the slope $c_{\mathrm{AI}}^{\mathrm{sim}}(\Delta Z)$ obtained from fitting the simulation data is also consistent with our theoretical prediction Eq. 4. The remaining shear modulus *G*_I_ = *G* − *G*_AI_ linearly depends on Δ*Z* (see Fig. 6A), with a *P*_0_-independent slope in agreement with the analytical result *c*_I_ = 1/30 as in the case of pure compression (7). Note that here Δ*Z* is increased by shear instead of compression. We find in our simulations that the pressure *P* does not depend on *μ*, but solely on Δ*Z*. Thus, the constant-*Z* ensemble considered here is in fact equivalent to a constant-*P* ensemble.

Our results show that, within the first-order approximation, the excess coordination number and anisotropy make additive contributions to the shear modulus. At a large strain, the contact network becomes strongly anisotropic, in which case the anisotropic correction to *G* is important. On the other hand, in the limit *σ* → 0 (or *γ* → 0), the anisotropic contribution is subleading: combining the scalings Δ*Z* ∼ *σ*^2/5^ and *μ* ∼ *σ*^1/4^ (Fig. 3B and C) with Eqs. 1, 2, 3, one obtains *G* ∼ Δ*Z* ∼ *σ*^2/5^ (Fig. 3A).

**Fig. 8.**
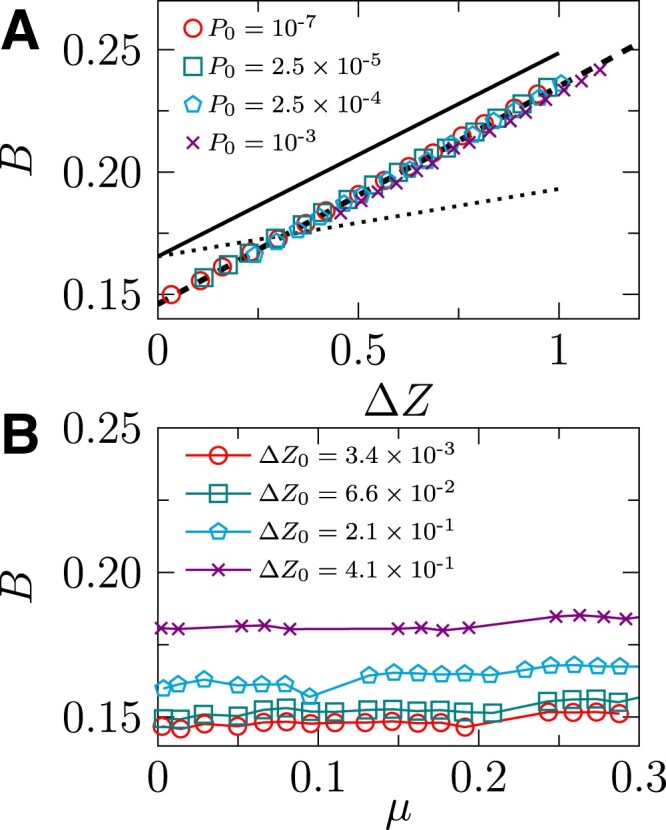
Bulk modulus of simulated systems. A) Bulk modulus *B* of strained configurations as a function of Δ*Z* for several *P*_0_. The dashed line represents the linear fitting, *B* = 0.147 + 0.0896Δ*Z*. The solid and dotted lines are Eq. 5 and Reuss estimation with *c*_0_ ≈ 0.17. B) Bulk modulus *B* of samples with a varying *μ*. Each curve has a constant Δ*Z* = Δ*Z*_0_, which is realized by decompression as in Fig. 6 (see the legend in A) for the corresponding *P*_0_ values).

For comparison, we also derive a theoretical expression of the bulk modulus *B* near the jamming transition (see Supplementary Material, Sec. 6),


(5) $$B=c_{0}+\left(\frac{1}{18}+\frac{c_{0}}{6}\right)\Delta Z,$$

where the constant $c_{0}=\frac{\langle f\rangle^{2}}{3\langle f^{2}\rangle }$ is identical to the one appeared in Eq. 4 for the shear modulus. In the jamming limit Δ*Z* → 0, the bulk modulus is a constant *B*(Δ*Z* = 0) = *c*_0_. In other words, *B* changes discontinuously at either compression or shear jamming, following a scaling *B* ∼ Δ*Z*^0^ as previously reported (2, 16). Equation (5) further gives the next-order correction that depends linearly on Δ*Z*. Plugging *c*_0_ ≈ 0.17 into Eq. 5 gives *B* ≈ 0.17 + 0.084Δ*Z*, which is close to the result *B* = 0.146 + 0.0897Δ*Z* obtained from fitting the simulation data (see Fig. 8A). As expected and suggested by Eq. 5, the bulk modulus *B* is independent of the anisotropy *μ* (see Fig. 8B for the numerical verification).

In our theoretical analysis, the internal stresses is ignored. Because the considered interaction is purely repulsive, the non-zero internal stress should lower the energy and therefore the modulus. In other words, the modulus obtained from the current theory overestimates the actual value. Approaching the jamming transition where the internal stress vanishes, the current theoretical results are very close to the simulation data, suggesting that the effects of internal stress are not significant in this limit (see Figs. 6 and 8).

Interestingly, a previous study (50) provided an approximated expression $B^{\mathrm{Reuss}}=\frac{Z\langle f\rangle^{2}}{18\langle f^{2}\rangle }=\frac{Zc_{0}}{6}$ for the bulk modulus using the so-called Reuss estimate (51). In Fig. 8, we compare *B*^Reuss^ with our theory Eq. 5 and simulation data. To make a further comparison, we expand *B*^Reuss^ to the linear order of Δ*Z*, using *Z* ≈ 6 + Δ*Z*, which results in $B_{}^{\mathrm{Reuss}}\approx c_{0}+\frac{c_{0}}{6}\Delta Z$. Compared to our theoretical result Eq. 5, this final expression gives the same constant *c*_0_ when Δ*Z* → 0, but a different coefficient of the linear term.

## Discussion

Our finding raises several challenges to the theoretical understanding of jamming transition and rheology of amorphous solids. In particular, the scaling *μ* ∼ *σ*^*β*^ with β* ≈ 0.25, which is universal in two to four dimensions (see Supplementary material), requires an quantitative theoretical explanation. The scaling ansatz proposed in Ref. (4), $G=\Delta ZG_{0}(\frac{\Delta \varphi }{\Delta Z^{2}},\frac{\gamma }{\Delta Z^{3/2}},N\Delta Z)$, treats Δ*Z* as the only relevant order parameter for the jamming transition. Equation (2) suggests that the ansatz needs to be extended, since the anisotropic part *G*_AI_, which relies on *μ* instead of Δ*Z*, contributes independently to the total shear modulus.

The response of amorphous solids to compression and shear has been studied by a first-principle mean-field theory—the state-following glass theory (52, 53). In the stable-glass phase, the theoretical outcome is *σ* ∼ *Gγ* and *P* ∼ *P*_0_ + *Rγ*^2^, where *R* is the dilatancy parameter (note that the second relation reflects the symmetry *P*(*γ*) = *P*( − *γ*)). These relationships give a scaling *μ* ∼ *σ* in the limit *γ* → 0, which can only explain the first scaling (in the elastic and softening regimes) in Fig. 4B. The issue is two-fold: on the one hand, it remains technically difficult to perform the state-following computation in the marginally stable phase where jammed states belong to (53); on the other hand, conceptually one should consider the possibility of “state rejuvenation” since simulations suggest φ_j_ decreasing towards φ_J_ in the shear hardening regime (22, 38).

Finally, it would be interesting to examine the effect of friction on shear hardening. Recent progress in producing ultra-stable granular materials makes an experimental investigation of frictional shear hardening feasible (12, 54). It is expected that the critical exponents are non-universal with different friction coefficients (54). In addition, the correction to the isotropic shear modulus should be even more significant, because friction amplifies spatial heterogeneity and orientational anisotropy of the contact network.

## Materials and methods

### Simulation models

We consider a 3D granular model of frictionless poly-disperse spheres with a continuous diameter distribution *P*(*D*) ∼ *D*^−3^, for *D*_min_ ≤ *D* ≤ *D*_min_/0.45 (20, 43, 44). The particles interact via a harmonic SS pair potential, $v_{ij}(r_{ij})=\frac{1}{2}(1-r_{ij}/D_{ij}{)}^{2}$ (zero if *r*_*ij*_ > *D*_*ij*_), where *r*_*ij*_ is the inter-particle distance and *D*_*ij*_ = (*D*_*i*_ + *D*_*j*_)/2 the mean diameter of particles *i* and *j*. We set the average diameter $\bar{D}$ as the unit of length. The lowest jamming density, or the J-point density (2), of this model is φ_J_ ≈ 0.655 (20, 55). The reported results are obtained from systems of *N* = 2000 particles, averaged over 192 independent samples. In addition, we study 2D and 4D models with the same kind of interaction in Supplementary material.

### Preparation of initial states

Two approaches are used to prepare ultra-stable jammed configurations. (i) Swap thermal annealing. The thermal annealing is realized by applying an efficient swap Monte Carlo algorithm (43, 44), which generates equilibrium hard sphere (HS) configurations at φ_eq_ above the mode-coupling theory (MCT) crossover density φ_MCT_ ≈ 0.594 (56). After that, we set the temperature to zero in the remaining simulations, and switch to the SS potential *v*_*ij*_(*r*_*ij*_). By applying athermal quasi-static compressions (AQCs) with the FIRE energy minimization algorithm (57) as described in (21, 55, 58), we obtain jammed configurations at φ_eq_-dependent jamming densities φ_j_ (20, 55, 59). In the main text, we focus on the case of φ_j_ = 0.69 unless otherwise specified, corresponding to φ_eq_ = 0.643. (ii) Mechanical training. The method is realized by cyclic AQS (21, 22, 23, 60) or cyclic AQC (13). Random initial configurations are rapidly compressed over φ_J_, and become unjammed after a sufficient number of cyclic AQS or AQC under the constant-volume condition (21), meaning that the jamming density is increased to φ_j_ > φ_J_. In both approaches, φ_j_ correlates to the stability of the jammed states, and reflects the degree of annealing.

The above procedures generate packings at φ = φ_j_ and *P* = 6 × 10^−9^, which are extremely close to the jamming/unjamming transition. They are then compressed quasi-statically to over-jammed states at a series of pressures *P*_0_ ≡ *P*(*γ* = 0). The pressure *P*_0_ characterizes the distance to the jamming point, via the well-known scaling between *P*_0_ and the density φ_0_ for harmonic SSs (2), *P*_0_ ∼ (φ_0_ − φ_j_). In order to remove residual stresses, the shear stabilization method (61) is applied, and these configurations with zero residual stresses are referred to as the *unstrained states* (*σ* = 0). According to this definition, in finite-size unstrained states, the stress *σ* is always zero, while the strain *γ* can fluctuate around zero from sample to sample. In our scaling analysis (see Fig. 3), it is better to use *σ* than *γ* as the independent variable, since the latter can introduce additional uncertainties. Of course, in the limit of large sample size and/or of larger number of samples, this sample-to-sample fluctuation disappears. In fact, a similar issue exists in the case of compression jamming: the jamming transition is defined at the zero pressure, while the jamming density can fluctuate around the mean value in finite-size simulations. Thus practically it is better to use the pressure than the density as the independent variable in scaling analyses.

### Compression and shear protocols

Compression and decompression procedures are realized by rescaling particle diameters with energy minimization. Starting from the unstrained states, we apply constant-volume, simple AQS in the *x*-*z* plane (21, 20). At each step, the shear strain *γ* is increased by *δγ*, followed by the FIRE energy minimization, where *δγ* is logarithmically increased from 10^−8^ to 2 × 10^−4^.

The stress and pressure are calculated using the virial formula (62), $\sigma =-\frac{1}{N_{\mathrm{NR}}}\sum_{b=1}^{N_{b}}r_{b}^{x}f_{b}^{z}$ and $P=\frac{1}{3N_{\mathrm{NR}}}\sum_{b=1}^{N_{b}}{\vec{r}}_{b}\cdot {\vec{\;f}}_{b}$, where ${\vec{r}}_{b}$ and ${\vec{\;f}}_{b}$ are the contact vector and force on bond *b*, *N*_NR_ the number of non-rattler particles (rattlers have fewer than *d* + 1 contacts in *d* dimensions), and *N*_b_ the total number of bonds. The shear modulus is defined as *G* = 〈*δσ*/*δγ*〉 + 〈*P*〉, averaged over 192 independent samples, where *δγ* is between 10^−10^ and 10^−8^. The term *δσ*/*δγ* corresponds to the slope of a single-realization stress–strain curve, which is piecewise linear (63) (see the inset of Fig. 1B). The correction term 〈*P*〉, which is typically three orders smaller than the first term, is added so that *G* is equivalent to the *xzxz* component of the stiffness matrix. We have checked that the value of *G* obtained in this way is identical to that directly calculated using the explicit expressions derived from the elastic theory (34). The bulk modulus is computed by the formula $B=-\langle \delta P/\delta \epsilon_{V}\rangle -\frac{1}{3}\langle P\rangle$, where $\epsilon_{V}$ is the volumetric strain and $\delta \epsilon_{V}\leq 2\times 10^{-9}$.

The changes of two independent parameters *Z* and *μ* reflect local rearrangements of particles. The increase in the number of contacts is quantified by the excess coordination number, Δ*Z* = *Z* − *Z*_iso_, where *Z*_iso_ = 2*d* − 2*d*/*N*_NR_ is the coordination number required to satisfy the isostatic condition at the jamming transition in *d* dimensions with a finite-size correction (64, 65), and rattlers are excluded in the computation of *Z*. The macroscopic friction coefficient *μ* = *σ*/*P* measures the degree of anisotropy caused by shear (66).

## Supplementary Material

pgad047_Supplementary_Data

## Data Availability

All data are available in the main text or the supplementary information.
