# Supplementary material for: Shear hardening in frictionless amorphous solids near the jamming transition
Source: PNAS Nexus. 2023 Feb 10;2(3):pgad047. doi: 10.1093/pnasnexus/pgad047 (PMC9991460; doi:10.1093/pnasnexus/pgad047)
Supplement: pgad047_Supplementary_Data [file pgad047_supplementary_data.pdf]

Supporting Information for

Shear hardening in frictionless amorphous solids near the jamming transition

Deng Pan, Fanlong Meng and Yuliang Jin

Corresponding Author Yuliang Jin.
E-mail: yuliangjin@mail.itp.ac.cn

This PDF file includes:

Supporting text
Figs. S1 to S6
SI References

Supporting Information Text

1. Shear strain as the independent variable

In our simulations, the stress σ has much smaller fluctuations than the strain γ (see *Materials and Methods*). Thus σ is chosen as the independent variable in our scaling analyses (see Fig. 3). For completeness, in Fig. S1, we plot G, ΔZ, μ and P as functions of γ. The data are essentially equivalent to those presented in Fig. 3, with γ treated as the independent variable.

2. Independence of scaling exponents on the degree of annealing

In the main text, we show that shear hardening presents in deeply annealed packings ($\varphi_{\rm j} = 0.69$). Here, we perform additional simulations on systems with different $\varphi_{\rm j}$ (i.e., different degrees of annealing). The data in Fig. S2 show that the exponents in all three scalings, $G \sim \sigma^{2/5}$, $\Delta Z \sim \sigma^{2/5}$ and $\mu \sim \sigma^{1/4}$, are independent of $\varphi_{\rm j}$. Although the curves of different $\varphi_{\rm j}$ seem to collapse in the figure completely, one should note that the yielding stress vanishes ($\sigma_{\rm Y} \to 0$) in the limit of $\varphi_{\rm j} \to \varphi_{\rm J}$ (1), and consequently so does the scaling regime of shear hardening.

3. Shear hardening in systems prepared by mechanical training

Dense packings with a large $\varphi_{\rm j}$ can be generated by either thermal or mechanical annealing. The latter refers to athermal training procedures such as cyclic shear or cyclic compression. The simulation data presented in the main text are obtained from well-annealed systems prepared by swap thermal annealing. Here, we present the results of samples annealed by mechanical training, and show that the scaling exponents are independent of the annealing protocol.

Two different training methods, cyclic shear and cyclic compression, are used. In the cyclic shear protocol (2), random configurations are firstly over-compressed to a density $\varphi_x > \varphi_{\rm J}$. Then, multiple cycles of shear, $0 \to \gamma_{\rm max} \to -\gamma_{\rm max} \to 0$, are applied until the system becomes unjammed at φ_x. In the cyclic compression protocol (3), the random configurations are over-compressed to a pressure $P_{\rm max}$, and then are decompressed to $\varphi = 0.62$ that is lower than the jamming density. Such cycles are stopped when the desired jamming density $\varphi_{\rm j} = 0.663$ is reached. Both mechanical training procedures are performed under the athermal quasi-static condition. The strain increment is $\delta\gamma = \pm 10^{-3}$ and the density increment is $\delta\varphi = \pm 10^{-4}$.

The jamming density $\varphi_{\rm j}$ depends on the parameters $\gamma_{\rm max}$ and φ_x in the cyclic shear protocol, and $P_{\rm max}$ in the cyclic compression protocol (2, 3). In this study, we set $\gamma_{\rm max} = 0.05$ and $\varphi_x = 0.66$ in the former, and $P_{\rm max} = 10^{-2}$ in the latter. With these parameters, we obtain $\varphi_{\rm j} = 0.662$ and $\varphi_{\rm j} = 0.663$ respectively, both of which are above the J-point density $\varphi_{\rm J} \approx 0.655$.

These mechanically trained systems also show shear hardening behavior (Fig. S3), with scalings identical to those in Fig. 3, which are obtained from systems generated by swap thermal annealing. We thus conclude that the scaling laws of shear hardening are independent of the way of annealing.

4. Shear hardening in two and four dimensions

The systems are composed by $N = 4000$ (2D) and $N = 2000$ (4D) particles. The diameter distribution is $p(D) \sim D^{-d}$, for $D_{\rm min} \leq D \leq D_{\rm max}/0.45$. As in the 3D model, two particles interact with each other via a harmonic soft sphere potential $v_{ij}(r_{ij})$, if they are in contact. The mean diameter of all particles is set as the unit length. All particles have the same unit mass.

In 2D, we use the swap thermal annealing protocol to prepare equilibrium hard disk liquids of density $\varphi_{\rm eq} = 0.85$. After that, we switch to the soft potential, and compress the system to a jammed state of $\varphi_{\rm j} = 0.881$ (the J-point density is $\varphi_{\rm J} = 0.841$).

We find that it is very easy to observe shear hardening in 4D. Deep annealing is unnecessary– it is enough to perform only one compression-decompression cycle. The jamming density of the obtained samples is $\varphi_{\rm j} = 0.486$, which is only slightly larger than the the J-point density $\varphi_{\rm J} = 0.480$.

The scaling behavior in 2D and 4D is reported in Fig. S4, together with the data obtained in 3D. The scaling exponents in the shear hardening regime are nearly independent of the dimensionality d. This observation is consistent with the previous conjecture of upper critical dimension $d_{\rm u} = 2$ for the jamming transition (4, 5).

5. Finite-size effects

To check finite size effects, we consider systems of different N, with the jamming density $\varphi_{\rm j} = 0.69$ and the unstrained initial pressure $P_0 = 10^{-6}$ fixed. The stress-strain curves with different N nearly coincide with each other before yielding (see Fig. S5A). The yielding stress $\sigma_{\rm Y}$ dependents on N, satisfying $\sigma_{\rm Y}(N) - \sigma_{\rm Y}(N = \infty) \sim N^{-0.5}$ (see the inset of Fig. S5A). In Fig. S5B, we plot μ as a function of σ, and find that there is no noticeable finite size effects on the crossover stress $\sigma_{\rm h}$. Thus the shear-hardening regime $\sigma_{\rm h} < \sigma < \sigma_{\rm Y}$ becomes smaller in larger systems, but remains finite in the thermodynamic limit.

6. Elasticity theory

A. Setup. *Model.* The model consists of N soft spheres (rattlers are not considered in this theoretical model) interacting via a harmonic potential at zero temperature. The total energy is

$$E = \sum_{i<j} v_{ij}(r_{ij}), \qquad [1]$$

where (we set both the spring constant and particle diameter to be one)

$$v_{ij}(r_{ij}) = \frac{1}{2}(1 - r_{ij})^2. \quad [2]$$

The vector $\vec{r}_{ij} = \vec{r}_j - \vec{r}_i$ goes from particle i to j. We are interested in the response of the model to athermal quasi-static shear, meaning that the system is force balanced at each shear step. Without loss of generality, we only compute the $xzxz$ component $G = \mathcal{C}^{xz,xz}$ of the stiffness tensor $\mathcal{C}$ as the shear modulus.

The following approximations are used, which are non-essential for the problem under consideration. (i) In simulations, the interaction is purely repulsive, and therefore Eq. (2) is truncated for $r_{ij} > 1$. The theoretical potential is perfectly symmetric. Thus the amorphous solid is modeled by a spring network. (ii) The polydispersity is neglected. (iii) The pre-stress is ignored in the calculation of shear modulus.

Notations. To avoid confusion, we use letters with arrows for spatial vectors in three dimensions (e.g., $\vec{r}_i = \{r_i^x, r_i^y, r_i^z\}$), and bold letters for general vectors (e.g., $\mathbf{v}, \mathbf{\Xi}, \mathbf{f}, \ldots$). Roman letters $(i, j, \ldots)$ denote particle indices, and Greek characters $(\alpha, \beta, \ldots)$ denote cartesian components.

Angular averages. The orientation of any interaction bond can be denoted by a unit vector in three dimensions, $\vec{n} = \{\sin(\theta)\cos(\phi), \cos(\theta), \sin(\theta)\sin(\phi)\}$. For a simple shear deformation in the x-z plane, the bond angles are distributed according to a function,

$$\rho(\theta, \phi) = \frac{1}{4\pi} - \frac{R_\mathrm{A}}{2\pi}\sin(2\phi), \quad [3]$$

characterized by the fabric anisotropy parameter R_A that can be computed from the fabric tensor (6, 7). The fabric tensor is defined as,

$$\mathcal{R} = \frac{1}{N}\sum_{b=1}^{N_\mathrm{b}} \vec{n}_b \otimes \vec{n}_b, \quad [4]$$

where N_b is the total number of contacts, and $\otimes$ is a vector outer product. The coordination number Z is the sum of eigenvalues of $\hat{R}$, $Z = \lambda_1 + \lambda_2 + \lambda_3$, and the fabric anisotropy parameter R_A is defined as $R_\mathrm{A} = (\lambda_\mathrm{max} - \lambda_\mathrm{min})/Z$.

With Eq. (3), the average of any angular dependent quantity can be calculated using the formula,

$$\left\langle n^\alpha n^\beta \ldots n^\chi \right\rangle = \int_0^\pi \sin\theta d\theta \int_0^{2\pi} d\phi\, \rho(\theta,\phi) n^\alpha n^\beta \ldots n^\chi. \quad [5]$$

In particular,

$$\langle n^x n^z \rangle = -\frac{R_\mathrm{A}}{3}, \quad [6]$$

and

$$\langle n^x n^z n^x n^z \rangle = \frac{1}{15}, \quad [7]$$

which is independent of R_A and will be used below.

Operators. In Ref. (8), Wyart introduces two operators $\mathcal{S}$ and $\mathcal{T}$, which make conversions between particle-based vectors (size $3N$) and bond-based vectors (size N_b). We briefly review their definitions and properties.

The matrix $\mathcal{S}$ (a N_b by $3N$ matrix) converts a particle-based displacement field $\delta\mathbf{R}$ (a vector of size $3N$) to displacements $\delta\mathbf{r}$ along interaction bonds (a vector of size N_b):

$$\mathcal{S}\delta\mathbf{R} = \delta\mathbf{r}, \quad [8]$$

Explicitly, the element of $\mathcal{S}$ is

$$(\mathcal{S}_b)_i^\alpha = (\delta_{li} - \delta_{mi}) n_{lm}^\alpha. \quad [9]$$

Here the contact b is formed between particles m and l, and b determines the row index. The column index is determined by i and α. Plugging Eq. (9) into Eq. (8), one obtains,

$$\left(\delta\vec{R}_i - \delta\vec{R}_j\right) \cdot \vec{n}_{ij} = \delta r_{ij}, \quad [10]$$

whose geometric interpretation is transparent: it projects the relative displacement between i and j on to the direction of bond. On the other hand, $\mathcal{T}$ (a $3N$ by N_b matrix) converts forces $\mathbf{f}$ along bonds (a vector of size N_b) into the net forces $\mathbf{F}$ on particles (a vector of size $3N$):

$$\mathcal{T}\mathbf{f} = \mathbf{F}, \quad [11]$$

or equivalently,

$$\sum_{j=1}^{N} c_{ij} f_{ij} \vec{n}_{ij} = \vec{F}_i, \qquad [12]$$

where c is the contact matrix ($c_{ij} = 1$ if particles i and j are in contact, otherwise $c_{ij} = 0$). The matrix $\mathcal{T}$ is the transpose of $\mathcal{S}$,

$$\mathcal{T} = \mathcal{S}^{\mathrm{T}}, \qquad [13]$$

and their product is the Hessian matrix,

$$\mathcal{H} = \mathcal{T}\mathcal{S}. \qquad [14]$$

where $\mathcal{H}$ is defined as,

$$\mathcal{H}_{ij}^{\alpha\beta} = \frac{\partial^2 E}{\partial r_i^\alpha \partial r_j^\beta}. \qquad [15]$$

B. Decomposition of the shear modulus into affine and non-affine parts. A microscopic elasticity theory for amorphous solids has been recently developed (9–13), which decomposes the shear modulus into affine and non-affine parts,

$$G = G_{\mathrm{A}} - G_{\mathrm{NA}}. \qquad [16]$$

The first term is the seminal Born-Huang formula derived for lattices (14),

$$G_{\mathrm{A}} = \frac{1}{N} \sum_{b=1}^{N_{\mathrm{b}}} n_b^x n_b^z n_b^x n_b^z, \qquad [17]$$

considering only affine displacements of particles. Here $N_{\mathrm{b}} = NZ/2$ and $\vec{n}_b$ is the unit vector along the bond.

The term G_{NA}, which does not appear in lattices, originates from corrections caused by non-affine displacements that are necessary to satisfy the force balance conditions during shear,

$$G_{\mathrm{NA}} = \frac{1}{N} \sum_{k=1}^{3N} \frac{(\mathbf{\Xi} \cdot \mathbf{v}_k)(\mathbf{\Xi} \cdot \mathbf{v}_k)}{\lambda_k}. \qquad [18]$$

Here $\mathbf{\Xi}$ is the *affine force* (also called *mismatch force*) field acting on N particles; $\mathbf{v}_k$ and λ_k are respectively the k-th normalized eigenvector and eigenvalue of the Hessian matrix $\mathcal{H}$. Both $\mathbf{\Xi}$ and $\mathbf{v}_k$ are vectors of size $3N$, and there are in total $3N$ eigenvectors of the Hessian matrix. The affine force $\mathbf{\Xi}$ can be computed explicitly for the given energy Eqs. (1) and (2):

$$\Xi_i^{\alpha xz} = \frac{\partial^2 E}{\partial r_i^\alpha \partial \epsilon^{xz}} = -\sum_j c_{ij} n_{ij}^x n_{ij}^z n_{ij}^\alpha, \qquad [19]$$

where ϵ is the strain matrix.

C. Decomposition of the shear modulus into isotropic and anisotropic parts. A formally different expression of shear modulus is derived by Wyart (8), based on the duality between force propagation and soft modes. The total shear modulus is decomposed into isotropic and anisotropic parts,

$$G = G_{\mathrm{I}} + G_{\mathrm{AI}}, \qquad [20]$$

where

$$G_{\mathrm{I}} = \frac{1}{N} \sum_{b=1}^{N_{\mathrm{b}}} \sum_{p=1}^{\frac{1}{2}N\Delta Z} \tilde{f}_{p,b} \tilde{f}_{p,b} n_b^x n_b^z n_b^x n_b^z, \qquad [21]$$

and

$$G_{\mathrm{AI}} = \frac{1}{N} \sum_{b_1 \neq b_2}^{N_{\mathrm{b}}} \sum_{p=1}^{\frac{1}{2}N\Delta Z} \tilde{f}_{p,b_1} \tilde{f}_{p,b_2} n_{b_1}^x n_{b_1}^z n_{b_2}^x n_{b_2}^z. \qquad [22]$$

Here $\tilde{\mathbf{f}}_p$ is the p-th state of self stress, a normalized vector representing the set of interaction forces (along the bond directions) that can satisfy the force balance conditions on every particle. The $\frac{1}{2}N\Delta Z$ vectors of $\tilde{\mathbf{f}}_p$ are orthogonal to each other. Among them, only $\tilde{\mathbf{f}}_1$ corresponds to the real forces $\mathbf{f}$ generated in simulations, which have to be positive for all contacts:

$$\tilde{\mathbf{f}}_1 = \mathbf{f} \Big/ \left(\sum_b f_b^2 \right)^{1/2}. \qquad [23]$$

The rest of the vectors $\tilde{\mathbf{f}}_p$ with $p \geq 2$ are "virtual" forces, having a roughly equal number of positive and negative components (8), i.e.,

$$\sum_{b=1}^{N_b} \tilde{f}_{p,b} \approx 0 \qquad [24]$$

for $p \geq 2$.

Under the effective media approximation, i.e., assuming independence between forces and bond orientations, $\langle \tilde{f}_{p,b}\tilde{f}_{p,b} n_b^x n_b^z n_b^x n_b^z \rangle \approx \langle \tilde{f}_{p,b}\tilde{f}_{p,b}\rangle \langle n_b^x n_b^z n_b^x n_b^z \rangle$, Eq. (21) becomes

$$G_{\rm I} \approx c_{\rm I} \Delta Z = \frac{1}{30}\Delta Z, \qquad [25]$$

where we have used the orthonormality of $\tilde{\mathbf{f}}_p$ and Eq. (7). Note that Eq. (25) is independent of bond orientations (or the anisotropy parameter $R_{\rm A}$), thus capturing only isotropic contributions. Indeed, Eq. (25) agrees well with the shear modulus measured in isotropically compressed packings (13), but not in sheared packings (see Fig. 6A).

In Eq. (22), the term of positive real forces ($p = 1$) dominates; the positive and negative virtual forces are roughly equally probable and therefore the terms of virtual forces ($p \geq 2$) make minor contributions. The anisotropic shear modulus can then be approximately expressed as,

$$G_{\rm AI} \approx \frac{1}{N} \frac{\sum_{b_1 \neq b_2} f_{b_1} f_{b_2} n_{b_1}^x n_{b_1}^z n_{b_2}^x n_{b_2}^z}{\sum_b f_b^2}, \qquad [26]$$

where we have used Eq. (23).

D. Equivalence of two decompositions. Here we prove the equivalence between the above two decompositions, by deriving Eqs. (21) and (22) from Eqs. (17) and (18). Our derivation is based on the formalism developed in Ref. (8) The goal is to replace the set of eigenvectors $\mathbf{v}_k$, where $k = 1, 2, \ldots 3N$, in Eq. (18) by the set of equilibrium forces $\tilde{\mathbf{f}}_p$ that balance all particles, where $p = 1, 2, \ldots \frac{1}{2}N\Delta Z$. This can be done by imposing the conditions of mechanical equilibrium, which connects displacement fields to equilibrium forces.

The first step is to project the particle-based displacement fields $\mathbf{v}_k$ onto the directions of bonds, by applying Eq. (8):

$$\mathbf{v}_k^{\parallel} = \frac{1}{\sqrt{\lambda_k}} \mathcal{S} \mathbf{v}_k, \qquad [27]$$

where the pre-factor $\frac{1}{\sqrt{\lambda_k}}$ ensures the normalization. Plugging Eq. (9) into the above equation gives,

$$v_{k,b}^{\parallel} = \frac{1}{\sqrt{\lambda_k}} \sum_{\alpha} \left[(v_k)_l^{\alpha} - (v_k)_m^{\alpha} \right] n_{lm}^{\alpha}. \qquad [28]$$

Using this expression and Eq. (19), we can rewrite Eq. (18) as,

$$G_{\rm NA} = \frac{1}{N} \sum_{b_2=1}^{N_{\rm b}} \sum_{k=1}^{3N} \sum_{b_1=1}^{N_{\rm b}} v_{k,b_1}^{\parallel} v_{k,b_2}^{\parallel} n_{b_1}^x n_{b_1}^z n_{b_2}^x n_{b_2}^z. \qquad [29]$$

Second, based on the equilibrium condition $\mathcal{T}\tilde{\mathbf{f}}_p = 0$, it can be shown that, for any k and p, $\mathbf{v}_k^{\parallel}$ and $\tilde{\mathbf{f}}_p$ have to be perpendicular to each other. This is because,

$$\mathbf{v}_k^{\parallel} \cdot \tilde{\mathbf{f}}_p = \frac{1}{\sqrt{\lambda_k}} (\mathbf{v}_k)^{\rm T} \left(\mathcal{T} \tilde{\mathbf{f}}_p \right) = 0, \qquad [30]$$

where we have used Eqs. (13) and (27). Equation (30) guarantees the minimization of energy at equilibrium. If we further require that $\tilde{\mathbf{f}}_p$'s are perpendicular to each other, then $\mathbf{v}_k^{\parallel}$ and $\tilde{\mathbf{f}}_p$ form a complete orthonormal basis of the vector space of dimension $N_{\rm b}$ (note that in the jammed phase, $N_{\rm b} = 3N + \frac{1}{2}N\Delta Z$), which can be represented by an $N_{\rm b}$ by $N_{\rm b}$ orthonormal matrix,

$$\begin{pmatrix} v_{1,1}^{\parallel} & v_{1,2}^{\parallel} & \cdots & v_{1,N_{\rm b}}^{\parallel} \\ v_{2,1}^{\parallel} & v_{2,2}^{\parallel} & \cdots & v_{2,N_{\rm b}}^{\parallel} \\ \vdots & & & \\ v_{3N,1}^{\parallel} & v_{3N,2}^{\parallel} & \cdots & v_{3N,N_{\rm b}}^{\parallel} \\ \tilde{f}_{1,1} & \tilde{f}_{1,2} & \cdots & \tilde{f}_{1,N_{\rm b}} \\ \tilde{f}_{2,1} & \tilde{f}_{2,2} & \cdots & \tilde{f}_{2,N_{\rm b}} \\ \vdots & & & \\ \tilde{f}_{\frac{1}{2}N\Delta Z,1} & \tilde{f}_{\frac{1}{2}N\Delta Z,2} & \cdots & \tilde{f}_{\frac{1}{2}N\Delta Z,N_{\rm b}} \end{pmatrix}. \qquad [31]$$

Because the columns of an orthonormal matrix are also orthonormal, we get,

$$\sum_{k=1}^{3N} v_{k,b_1}^{\parallel} v_{k,b_2}^{\parallel} + \sum_{p=1}^{\frac{1}{2}N\Delta Z} \tilde{f}_{p,b_1}\tilde{f}_{p,b_2} = \delta_{b_1 b_2}. \quad [32]$$

Finally, combining Eq. (32) with Eqs. (16), (17) and (29), we obtain Eqs. (21) and (22).

E. Anisotropic shear modulus. Next, we relate the anisotropic shear modulus Eq. (26) to the macroscopic friction coefficient $\mu = \sigma/P$. Using the virial expressions of σ and P (see Materials and Methods), we obtain,

$$\begin{aligned}\mu^2 &= 9\frac{\sum_b (f_b n_b^x n_b^z)^2 + \sum_{b_1\neq b_2} f_{b_1} f_{b_2} n_{b_1}^x n_{b_1}^z n_{b_2}^x n_{b_2}^z}{\left(\sum_b f_b\right)^2} \\ &\approx 9\frac{\sum_{b_1\neq b_2} f_{b_1} f_{b_2} n_{b_1}^x n_{b_1}^z n_{b_2}^x n_{b_2}^z}{\left(\sum_b f_b\right)^2}.\end{aligned} \quad [33]$$

Note that the spatial correlation between forces is short-ranged (15), which means that the first term $\sum_b (f_b n_b^x n_b^z)^2 / \left(\sum_b f_b\right)^2$ is of order $O(1/N_{\rm b})$, while the second term is of order $O(1)$. With Eq. (33), the anisotropic shear modulus Eq. (26) can be written as

$$G_{\rm AI} \approx \frac{Z}{18}\frac{\langle f\rangle^2}{\langle f^2\rangle}\mu^2. \quad [34]$$

Our simulation results show that the force distribution $p(f)$ is independent of μ (see Fig. S6). From $p(f)$, it is easy to evaluate $\langle f\rangle^2/\langle f^2\rangle \approx 0.50$. In addition, $Z \approx 2d = 6$ near the jamming transition. With these values, when $\Delta Z \to 0$ we have $G_{\rm AI} \approx 0.17\mu^2$.

It is also possible to consider the contributions from virtual forces $\tilde{\mathbf{f}}_p$ for $p \geq 2$. In analogy with Eq. (33), we obtain,

$$\sum_{b_1\neq b_2}^{N_{\rm b}} \tilde{f}_{p,b_1}\tilde{f}_{p,b_2} n_{b_1}^x n_{b_1}^z n_{b_2}^x n_{b_2}^z \sim -\mu^2, \quad [35]$$

for $p \geq 2$, where we have assumed that $\langle \tilde{f}_{p,b_1}\tilde{f}_{p,b_2} n_{b_1}^x n_{b_1}^z n_{b_2}^x n_{b_2}^z\rangle \approx \langle \tilde{f}_{p,b_1}\tilde{f}_{p,b_2}\rangle\langle n_{b_1}^x n_{b_1}^z n_{b_2}^x n_{b_2}^z\rangle$ for any p. The minus sign in Eq. (35) comes from the property of virtual forces Eq. (24), according to which, $(\sum_b^{N_b} \tilde{f}_{p,b})^2 = \sum_b^{N_b} \tilde{f}_{p_b}^2 + \sum_{b_1\neq b_2}^{N_b} \tilde{f}_{p,b_1}\tilde{f}_{p,b_2} \approx 0$, and thus $\sum_{b_1\neq b_2}^{N_b} \tilde{f}_{p,b_1}\tilde{f}_{p,b_2} < 0$. Plugging Eq. (35) into Eq. (22) gives a negative correction term $\sim -\Delta Z\mu^2$ to the shear modulus Eq. (34). With this correction, $G_{\rm AI}$ becomes,

$$G_{\rm AI} = c_{\rm AI}(\Delta Z)\mu^2 = (c_0 - \alpha\Delta Z)\mu^2 \quad [36]$$

where $c_0 = \frac{1}{3}\frac{\langle f\rangle^2}{\langle f^2\rangle}$ and α is a constant that can be determined from the fit of simulation data (see the inset of Fig. 6B). Equation (36) shows that the contribution of virtual forces is higher-order, which only appears in systems above jamming ($\Delta Z > 0$).

F. Bulk modulus. It is straightforward to generalize the theoretical analysis to other components of the stiffness tensor, $\mathcal{C}^{\alpha\beta,\gamma\delta}$. Similar to the above derivation, any component $\mathcal{C}^{\alpha\beta,\gamma\delta}$ can be decomposed into isotropic and anisotropic parts. The isotropic part is

$$\mathcal{C}_{\rm I}^{\alpha\beta,\gamma\delta} \approx \frac{1}{2}\langle n_b^\alpha n_b^\beta n_b^\gamma n_b^\delta\rangle\Delta Z, \quad [37]$$

which is a generalized formula of Eq. (25), and the anisotropic part is

$$\mathcal{C}_{\rm AI}^{\alpha\beta,\gamma\delta} = \frac{1}{N}\sum_{b_1\neq b_2}^{N_{\rm b}}\sum_{p=1}^{\frac{1}{2}N\Delta Z} \tilde{f}_{p,b_1}\tilde{f}_{p,b_2} n_{b_1}^\alpha n_{b_1}^\beta n_{b_2}^\gamma n_{b_2}^\delta, \quad [38]$$

which is a generalized formula of Eq. (22). In analogy with the treatment of the shear modulus, we can relate the anisotropic part $\mathcal{C}_{\rm AI}^{\alpha\beta,\gamma\delta}$ to the generalized stress anisotropy, $\mu_{\alpha\beta} = \sigma_{\alpha\beta}/P$, where $\sigma_{\alpha\beta}$ is the $\alpha\beta$-component of stress tensor,

$$\mathcal{C}_{\rm AI}^{\alpha\beta,\gamma\delta} = c_{\alpha\beta,\gamma\delta}(\Delta Z)\mu_{\alpha\beta}\mu_{\gamma\delta}, \quad [39]$$

where $c_{\alpha\beta,\gamma\delta}(\Delta Z = 0) = c_0$ and the sub-leading correction is proportional to ΔZ. In particular, the bulk modulus B is,

$$B = \frac{1}{9}\sum_\alpha\sum_\beta \mathcal{C}^{\alpha\alpha,\beta\beta} \approx c_0 + \left(\frac{1}{18} + \frac{c_0}{6}\right)\Delta Z \quad [40]$$

which is independent of the anisotropy.

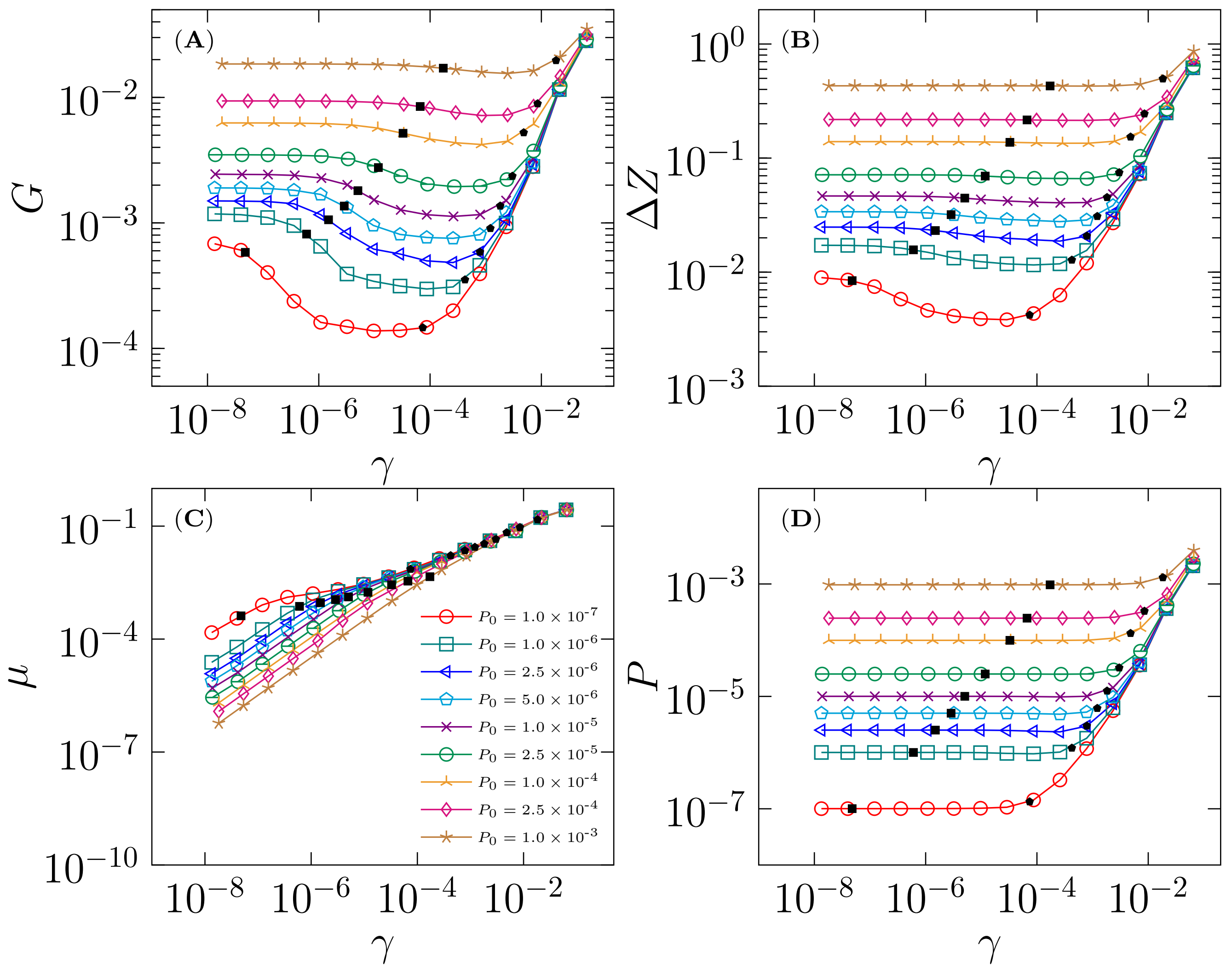

Fig. S1. Simulation results of (A) the shear modulus G, (B) the excess coordination number ΔZ, (C) the macroscopic friction coefficient μ and (D) the pressure P as functions of strain γ. The crossovers $\gamma_{\rm s}$ and $\gamma_{\rm h}$ are represented by solid squares and pentagons, respectively.

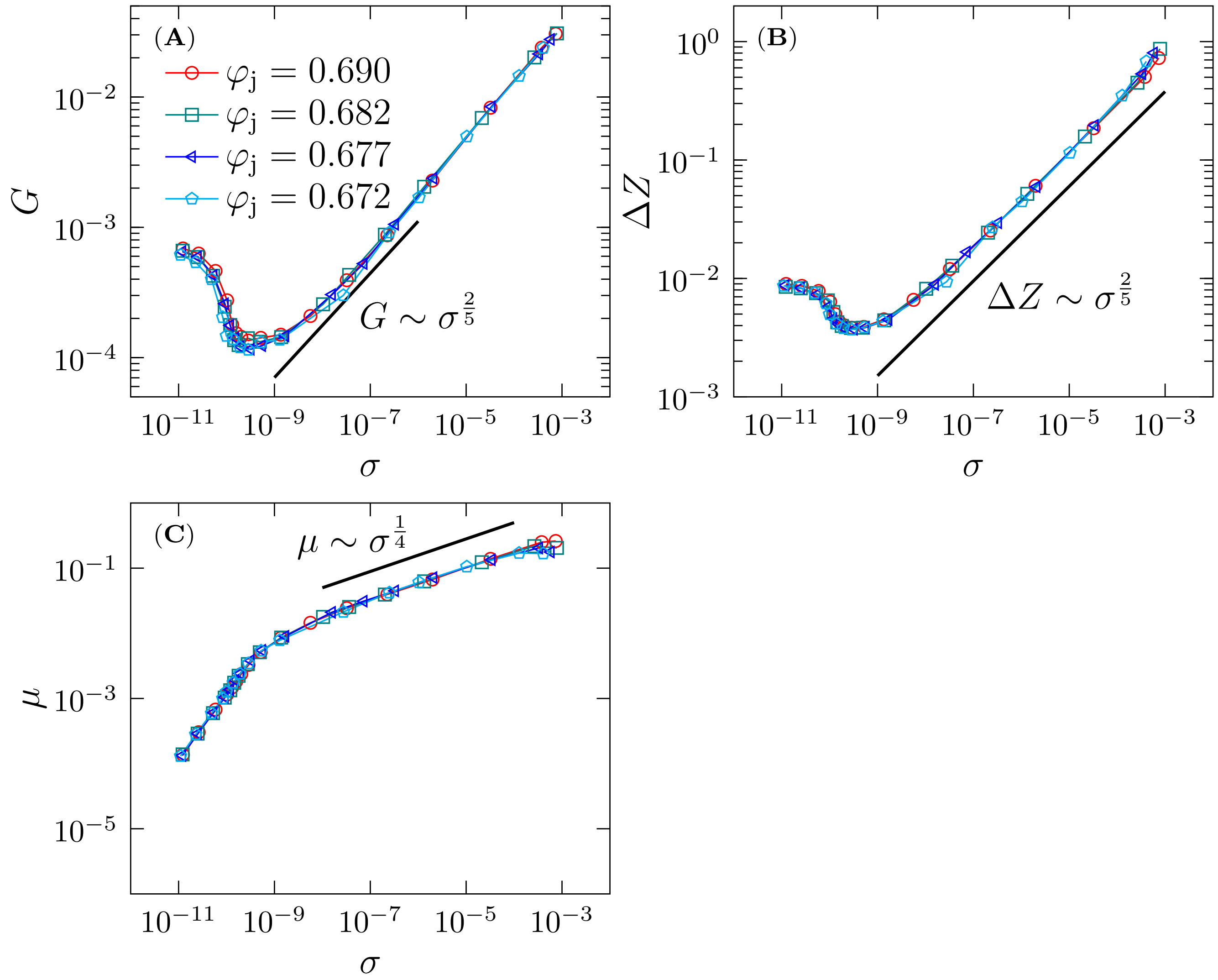

Fig. S2. Shear hardening scalings of systems with different degree of annealing. Simulation results of (A) shear modulus G, (B) excess coordination number ΔZ and (C) macroscopic friction coefficient μ, as functions of stress σ, for a few different $\varphi_{\rm j}$. The unstrained pressures are the same, $P_0 = 10^{-7}$. The solid lines represent scaling laws in the shear hardening regime, $G \sim \sigma^{2/5}$, $\Delta Z \sim \sigma^{2/5}$ and $\mu \sim \sigma^{1/4}$.

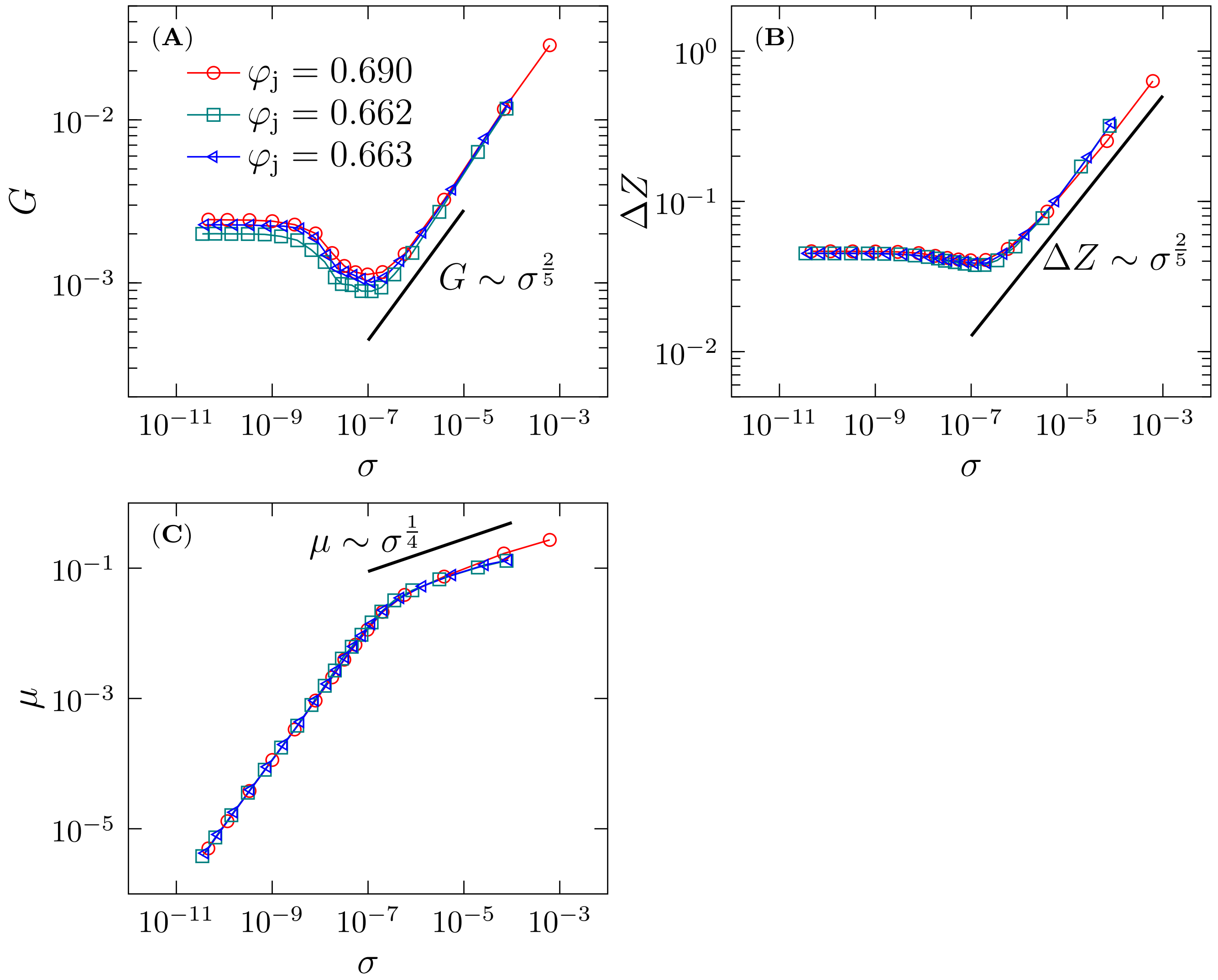

Fig. S3. Shear hardening scalings of systems prepared by thermal annealing and mechanical training. Simulation results of (A) shear modulus G, (B) excess coordination number ΔZ and (C) macroscopic friction coefficient μ, as functions of stress σ, for systems prepared by swap thermal annealing ($\varphi_{\rm j} = 0.69$), cyclic shear ($\varphi_{\rm j} = 0.662$) and cyclic compression ($\varphi_{\rm j} = 0.663$). The unstrained pressures are the same, $P_0 = 10^{-5}$.

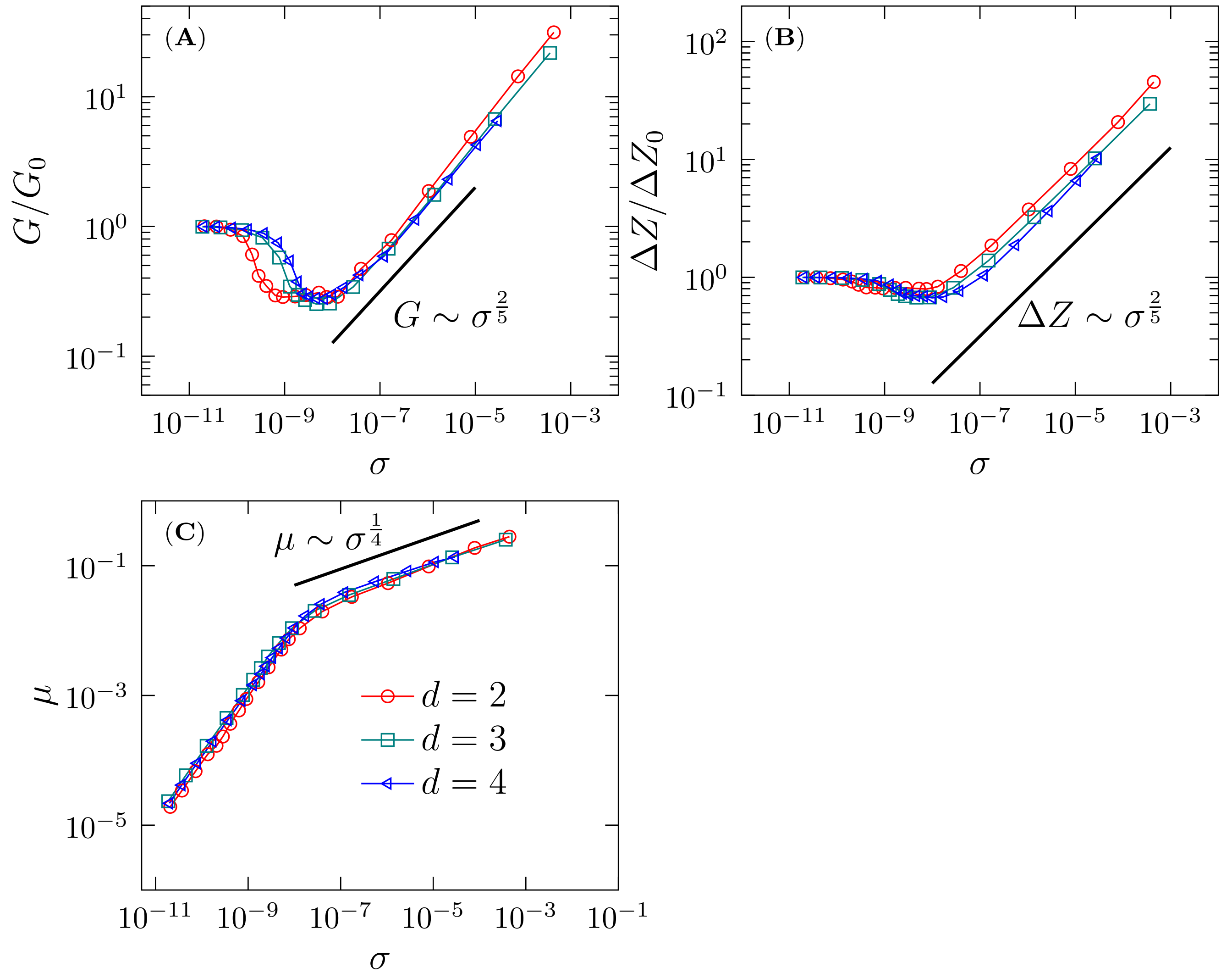

Fig. S4. Shear hardening scalings of systems in two to four dimensions. Simulation results of (A) shear modulus G, (B) excess coordination number ΔZ and (C) macroscopic friction coefficient μ versus stress σ in 2D ($\varphi_{\rm j} = 0.881$), 3D ($\varphi_{\rm j} = 0.690$) and 4D($\varphi_{\rm j} = 0.486$), for $P_0 = 10^{-6}$. In (A) and (B), G and ΔZ are scaled by their unstrained values G_0 and ΔZ_0 respectively.

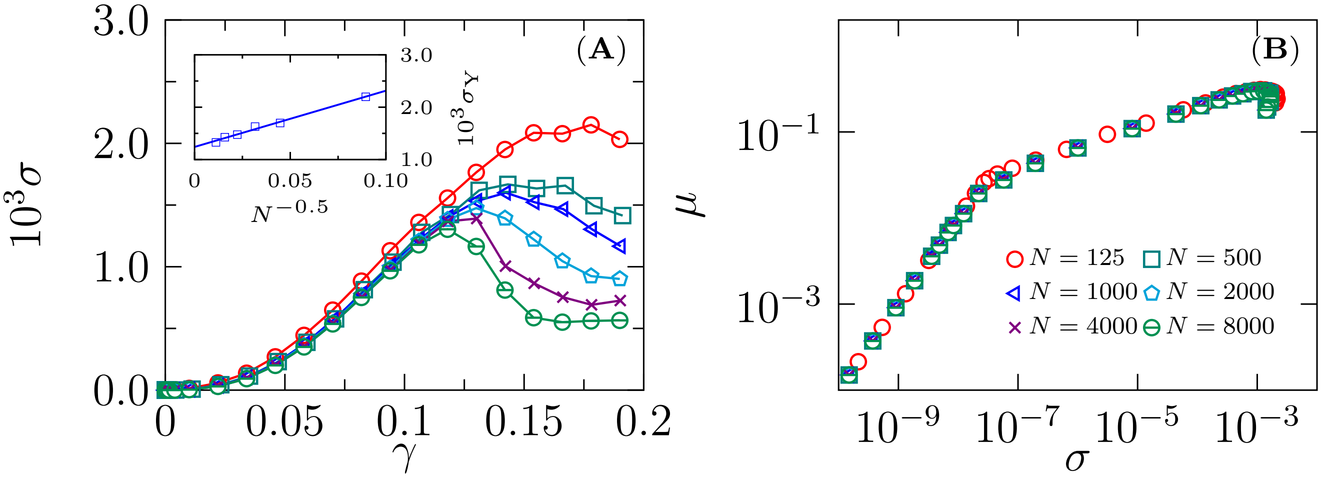

Fig. S5. (A) Stress-strain curves and (B) macroscopic friction coefficient μ as a function of stress σ for different system sizes N, with fixed $P_0 = 10^{-6}$ and $\varphi_{\rm j} = 0.69$. The system size dependence of yielding stress $\sigma_{\rm Y}$ (i.e., the maximum stress) is shown in the inset of (A) and the solid line is the fitting curve $\sigma_{\rm Y}(N) = 0.0107N^{-0.5} + 0.00124$.

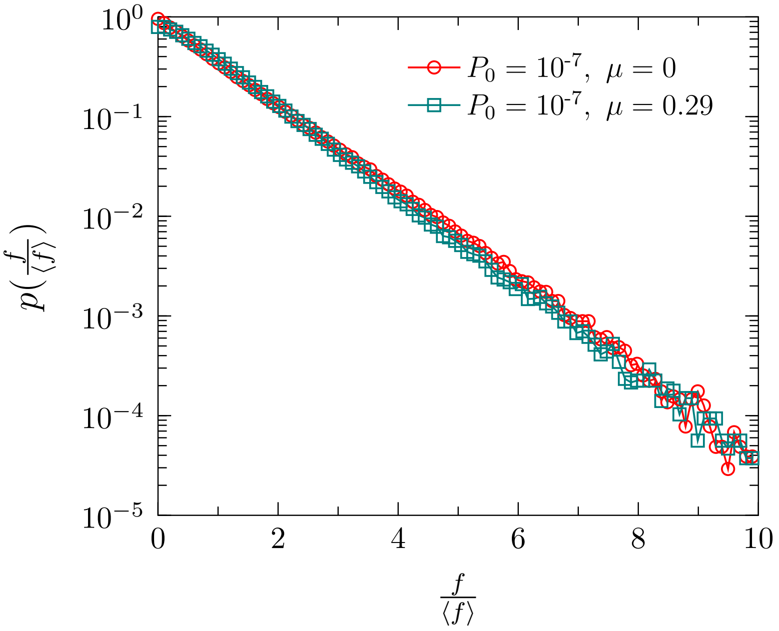

Fig. S6. Distribution of contact force in isotropic and anisotropic systems. The force distribution functions of isotropic ($P_0 = 10^{-7}, \mu = 0$) and sheared systems ($P_0 = 10^{-7}, \mu = 0.29$).

References

1. D Pan, T Ji, M Baggioli, L Li, Y Jin, Nonlinear elasticity, yielding, and entropy in amorphous solids. *Sci. Adv.* **8**, eabm8028 (2022).
2. V Babu, D Pan, Y Jin, B Chakraborty, S Sastry, Dilatancy, shear jamming, and a generalized jamming phase diagram of frictionless sphere packings. *Soft Matter* **17**, 3121–3127 (2021).
3. N Kumar, S Luding, Memory of jamming–multiscale models for soft and granular matter. *Granul. Matter* **18** (2016).
4. AJ Liu, SR Nagel, The jamming transition and the marginally jammed solid. *Annu. Rev. Condens. Matter Phys.* **1**, 347–369 (2010).
5. CP Goodrich, AJ Liu, SR Nagel, Finite-size scaling at the jamming transition. *Phys. Rev. Lett.* **109**, 095704 (2012).
6. F Radjai, DE Wolf, M Jean, JJ Moreau, Bimodal character of stress transmission in granular packings. *Phys. Rev. Lett.* **80**, 61–64 (1998).
7. Y Jin, H Yoshino, A jamming plane of sphere packings. *Proc. Natl. Acad. Sci.* **118**, e2021794118 (2021).
8. M Wyart, On the rigidity of amorphous solids. *Ann. Phys. Fr.* **30**, 1–96 (2005).
9. C Maloney, A Lemaitre, Universal breakdown of elasticity at the onset of material failure. *Phys. Rev. Lett.* **93**, 195501 (2004).
10. A Lemaître, C Maloney, Sum rules for the quasi-static and visco-elastic response of disordered solids at zero temperature. *J. Stat. Phys.* **123**, 415–453 (2006).
11. S Karmakar, E Lerner, I Procaccia, Athermal nonlinear elastic constants of amorphous solids. *Phys. Rev. E* **82**, 9 (2010).
12. A Zaccone, JR Blundell, EM Terentjev, Network disorder and nonaffine deformations in marginal solids. *Phys. Rev. B* **84**, 174119 (2011).
13. A Zaccone, E Scossa-Romano, Approximate analytical description of the nonaffine response of amorphous solids. *Phys. Rev. B* **83**, 184205 (2011).
14. M Born, K Huang, Dynamical theory of crystal lattices oxford university press. *London, New York* (1954).
15. CS O'Hern, LE Silbert, AJ Liu, SR Nagel, Jamming at zero temperature and zero applied stress: the epitome of disorder. *Phys. Rev. E* **68**, 011306 (2003).
